# Depressive Symptoms: The Interaction between Rumination and Self-Reported Insomnia

**DOI:** 10.1155/2015/150828

**Published:** 2015-11-04

**Authors:** Monique Malmberg, Junilla K. Larsen

**Affiliations:** Behavioural Science Institute, Radboud University Nijmegen, P.O. Box 9140, 6500 HE Nijmegen, Netherlands

## Abstract

*Objective*. Prior research has found consistent support that rumination and insomnia are important risk factors for depressive symptoms. The aim of the present cross-sectional study is to examine the interaction between these two previously well-established risk factors (i.e., rumination and insomnia) in the explanation of depressive symptoms. *Design*. A total of 417 participants (277 women) with a mean age of 39 (SD = 17.59; range 18–85) completed a cross-sectional survey. *Main Outcome Measures*. Participants filled out the Response Rumination Scale, the Athens Insomnia Scale, and the short version of the Center for Epidemiologic Studies Depression Scale. *Results*. It was predicted and found that self-reported insomnia moderated the relationship between rumination and depressive symptoms. We found that particularly participants who reported higher levels of rumination as well as insomnia had the highest depressive symptoms. *Conclusion*. This study is the first to suggest that particularly individuals exhibiting both self-reported insomnia and higher levels of rumination also report higher levels of depressive symptoms. Health professionals screening for mental problems should be aware of this specific combination of insomnia and rumination. Explanations for this moderation effect were discussed in light of study's limitations.

## 1. **Introduction**

Rumination is generally considered to be a maladaptive emotion regulation strategy and a major aetiological factor explaining the onset of depression [1]. It concerns a repetitive and passive focus on one's symptoms of distress and their possible causes and consequences. In the same line, a review of longitudinal epidemiological studies supports the link between insomnia and the development of depressive symptoms and depression [2]. Although both rumination and insomnia are considered well-established risk factors for the onset of depression, their potential combined influence has not been studied. Information on the different interactions among previously well-established risk factors is important for improving the understanding of the aetiology of the disorder [3]. We propose that specifically the combination of rumination* and* insomnia relates to higher levels of depressive symptoms.

One potential mechanism that might explain why this specific combination of rumination and insomnia renders one's susceptibility to the development of depressive symptoms is that individuals reporting both rumination and insomnia stay fixated on their feelings even when wanting or needing sleep. This fixation might reflect underlying psychological inflexibility, an important construct in explaining less optimal emotion regulation, making these individuals more susceptible to the development of depressive symptoms [4]. To the best of our knowledge, there is no information on the combination of rumination and insomnia in explaining psychological inflexibility. However, both rumination and insomnia have been associated with disturbances in cognitive functioning, including working memory and attentional inhibition, that are related to psychological inflexibility [5, 6]. Thus, individuals who report both rumination and insomnia might be particularly sensitive for the development of depressive symptoms, due to problems in flexible cognitive functioning.

Hence, the aim of the present cross-sectional study is to examine the interaction between rumination and self-reported insomnia in the explanation of depressive symptoms. We expect that self-reported insomnia moderates the association between rumination and depressive symptoms, with individuals exhibiting both rumination and self-reported insomnia reporting the highest levels of depressive symptoms. Although prospective research offers more insight in the causal order of associations, our study is the first to examine the combined influence of rumination and self-reported insomnia, making this study unique and relevant.

## 2. Method

### 2.1. Participants

A total of 417 Dutch adults (134 males and 277 females, 6 unknown) participated in the current study. The age of the participants ranged from 18 to 85 years (*M* = 38.99, SD = 17.59) and participants were primarily Caucasian (95%). About 70% of the participants were highly educated; they completed higher general or preuniversity training (27.5%), higher vocational training (26.2%), or university (16.6%).

### 2.2. Procedure

Research assistants (i.e., 12 bachelor and 3 master students) recruited adult participants in their neighbourhood. Each assistant asked adult (i.e., aged 18 or older) family members, friends, or acquaintances to fill out a questionnaire on emotions and health. It was emphasized that participation was voluntary. Data were collected anonymously. Some data collection also occurred through a snowball effect (with participating persons also spreading questionnaires). Approval was received from the Ethics Committee of the Faculty of Social Sciences of the Radboud University Nijmegen. After agreement, participants received the questionnaire in an envelope of the Radboud University of Nijmegen. The participants were instructed to seal the envelope after filling out the questionnaire and to return the sealed envelope to the university or the research assistant. Completing the questionnaire took approximately 30 minutes. Anonymity was guaranteed by sealing the envelopes and all data were entered in SPSS by a different research assistant than the recruiting assistant.

### 2.3. Measures

To measure rumination, a short version of the Ruminative Response Scale (RRS [7]) was used. This shortened version consists of 10 items rated on a 4-point scale ranging from (1) almost never to (4) almost always. Self-reported insomnia was assessed by means of the Athens Insomnia Scale (AIS [8]). The AIS is a self-assessment psychometric instrument designed for quantifying sleep difficulty based on the ICD-10 criteria. It consists of eight items and each item was rated on a 4-point scale, with (0) corresponding to “no problem at all” and (3) to “very serious problem.” Participants were asked to answer each item only if they experienced that particular sleep difficulty at least three times a week during the last month. The total AIS scores were skewed (skewness = 1.27, SE = 0.12) and revealed a distinct cut-off between 4 and 6. Hence, participants were classified as insomniac when they had an AIS score of 6 or higher and as subthreshold insomniac when they had an AIS score of 5 [9, 10]. A validated short form (i.e., Iowa form) of the Center for Epidemiological Studies Depression Scale (CES-D Scale [11]) was used to measure depressive symptoms. It consists of eleven items that were rated on a 4-point scale ranging from (0) never/seldom to (3) mostly/always. Cronbach's alpha for the CES-D was .82.

### 2.4. Analytic Strategy

We checked our data on normality, linearity, and homoscedasticity and calculated descriptive statistics. As reported, only scores on the AIS were skewed. Education, age, and gender were not significantly correlated with depressive symptoms in the total group (all *p*'s > .10), so we did not control for them in all subsequent analysis (further examination showed that gender differences were absent for individuals who completed either higher general or preuniversity training, or university (with men even displaying higher levels of depression, though not significant). We did find that lower educated women (but also women with higher vocational training) showed higher levels of depressive symptoms compared to men, a pattern in line with previous research). We performed hierarchical regression analyses to examine whether depressive symptoms were explained by the interaction of rumination and self-reported insomnia. In the first step, main effects of rumination and self-reported insomnia were entered. In the second step, we entered the interaction effect between rumination and self-reported insomnia. Prior to the regression, we centered rumination on the grand mean and dummy coded self-reported insomnia (0 = no; 1 = yes). All analyses were performed using the statistical software package SPSS 21.

## 3. Results

### 3.1. Background

Participants had an average sum score of 17.50 (SD = 4.75, range 10–35) on the short form of the RSS (10 items) and an average sum score of 5.15 (SD = 4.45, range 0–27) on the IOWA form of the CES-D (11 items). We recalculated the sum scores on the CES-D 11 Iowa version (with original anchors of the CES-D 20) to fit the original 20-item version to facilitate comparison with previous research. An average sum score of 9.36 was found (SD = 8.06, range 0–49). A total of 30.8% participants (*n* = 126; 8 missing cases) were classified insomniac according to a 6-point cut-off on the AIS, and an additional 6.9% were classified as having subthreshold insomnia. Positive correlations (all *p*'s < .0001) were found between rumination and depressive symptoms (*r* = .55), self-reported insomnia and depressive symptoms (*r* = .44), and rumination and self-reported insomnia (*r* = .29).

### 3.2. Main Analyses

**Figure 1:**
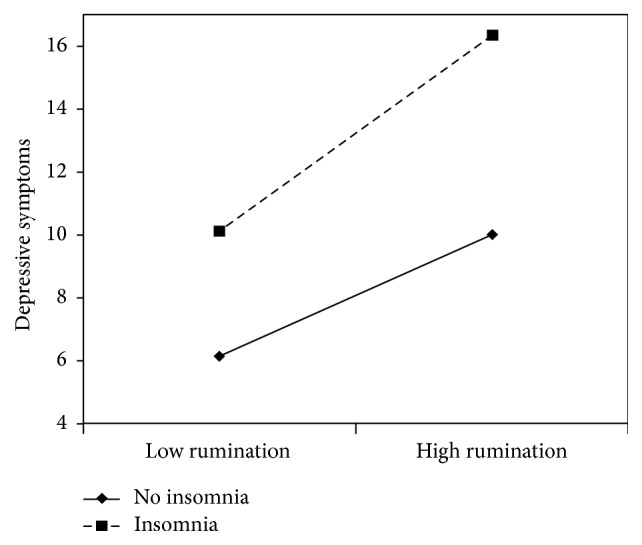
Moderating role of self-reported insomnia in the relationship between rumination and depressive symptoms.

Table 1 shows the results of the regression analysis examining the role of self-reported insomnia in the relationship between rumination and depressive symptoms. Both rumination and self-reported insomnia independently contributed to the explanation of depressive symptoms, together explaining nearly 40% of the variance in depressive symptoms. Simple slope analyses were calculated for values of insomnia scores ±1 SD. These analyses revealed significant regression lines for both individuals without (*β* = .13, *p* < .01) and with (*β* = .18, *p* < .01) self-reported insomnia. In addition, we found a significant interaction between rumination and self-reported insomnia (*β* = .14, *p* = .007). This interaction is depicted in Figure 1, showing a stronger positive relationship between rumination and depressive symptoms for participants with self-reported insomnia compared to those without self-reported insomnia. Figure 1 suggests that especially the combination of rumination and self-reported insomnia is associated with higher depressive symptoms. A similar interaction effect was found for self-reported subthreshold insomnia following a cut-off of 5 (*β* = .19, *p* < .0001; Δ*R* ^2^ = .02). No significant interactions of gender and education with rumination and self-reported insomnia diagnosis were found.

## 4. Discussion

Previous research has found consistent support that rumination and insomnia are important independent risk factors for depression [1, 2]. Our study adds to the field that a combination of these well-established risk factors might better explain depressive symptoms. It was hypothesized and found that self-reported insomnia moderated the relationship between rumination and depressive symptoms. We found that particularly participants who reported higher levels of rumination as well as self-reported insomnia had the highest depressive symptoms. In line with this finding, one previous study found that especially evening rumination relates to higher levels of depression [12]. It might be that individuals who experience both rumination and insomnia ruminate especially in the evening, instead of sleeping, and typically have difficulties relinquishing thought control when trying to fall asleep. Moreover, these individuals might exhibit less psychological flexibility to change these patterns (although they might want to). This psychological inflexibility might consequently make these individuals more susceptible to the development of depressive symptoms [4]. Future research should examine the role of psychological inflexibility in explaining patterns of rumination over the day and associations with insomnia and depressive symptoms.

Our study has some general limitations and related implications for future research. First, we are aware of the limitations with regard to the cross-sectional nature of our study. Nevertheless, our sample size is quite large and the interaction effect is robust and consistent. Our cross-sectional design prohibits drawing causal inferences. It is, for instance, possible that participants already suffered from depression and have developed comorbid insomnia. Future longitudinal research assessing rumination, insomnia, and depression multiple times will enhance the present insight on the directionality of the relationships found. Also, this will make it possible to test potential mediating models (e.g., rumination-insomnia-depression), considering the reciprocal associations found between all constructs [1, 2, 13]. Second, we did not ask participants what they were ruminating about. It is possible that individuals with insomnia were ruminating about their sleeping problems [14, 15], because they experienced more severe consequences of their insomnia compared to insomniacs with lower ruminative tendencies. Future prospective research can examine this and may take into account diurnal patterns of rumination. Finally, our participants were primarily highly educated. Findings from the present study cannot readily be generalized to lower educated populations, even though we were not able to find different patterns for the lower educated people in our study. The absence of gender differences among some educational groups is probably due to a more socially homogeneous sample of men and women [16] or the fact that male participants are part of the recruiting assistants' social network [17].

In sum, our findings indicate that particularly individuals exhibiting both self-reported insomnia and higher levels of rumination also report higher levels of depressive symptoms. Health professionals screening for mental problems should be aware of this specific combination of insomnia and rumination. In addition, incorporating a focus on both the improvement of sleep and the thinking patterns could enhance depression prevention programs. Yet, before setting up such a prevention trial, prospective studies are needed on the combined effect of self-reported insomnia and rumination on the development of depressive symptoms.

## Figures and Tables

**Table 1:** Hierarchical regression analysis of self-reported insomnia as a moderator in the relationship between rumination and depressive symptoms (*n* = 407).

	Depressive symptoms		
	B	SE B	*β*
Step 1			
Rumination	2.06	.18	.46^*∗∗*^
Insomnia	2.92	.39	.30^*∗∗*^
Step 2			
Rumination	1.71	.22	.38^*∗∗*^
Insomnia	2.68	.40	.28^*∗∗*^
Rumination *∗* Insomnia	1.03	.38	.14^*∗*^

*Note*. *R* ^2^ = .39 for Step  1 (*p* < .0001); Δ*R* ^2^ = .01 for Step  2 (*p* < .01).

^*∗*^ *p* < .01; ^*∗∗*^ *p* < .0001.
